# Effects of Tissue Pressure on Transgene Expression Characteristics via Renal Local Administration Routes from Ureter or Renal Artery in the Rat Kidney

**DOI:** 10.3390/pharmaceutics12020114

**Published:** 2020-02-01

**Authors:** Natsuko Oyama, Haruyuki Takahashi, Maho Kawaguchi, Hirotaka Miyamoto, Koyo Nishida, Masako Tsurumaru, Mikiro Nakashima, Fumiyoshi Yamashita, Mitsuru Hashida, Shigeru Kawakami

**Affiliations:** 1 Graduate School of Biomedical Sciences, Nagasaki University, 1-7-1 Sakamoto, Nagasaki-shi, Nagasaki 852-8588, Japan; n.oyama.nagasaki.u@gmail.com (N.O.); bb55319402@ms.nagasaki-u.ac.jp (M.K.); hmiyamoto@nagasaki-u.ac.jp (H.M.); koyo-n@nagasaki-u.ac.jp (K.N.); masakot@nagasaki-u.ac.jp (M.T.); mikirou@nagasaki-u.ac.jp (M.N.); 2 Graduate School of Pharmaceutical Sciences, Kyoto University, 46-29 Yoshida-Shimo-Adachi-cho, Sakyo-ku, Kyoto 606-8501, Japan; kuph04@gmail.com (H.T.); yamashita.fumiyoshi.3m@pharm.kyoto-u.ac.jp (F.Y.); hashidam@pharm.kyoto-u.ac.jp (M.H.)

**Keywords:** naked pDNA, physical methods, pressure, gene transfection, kidney, local administration, renal artery, renal ureter

## Abstract

We previously developed a renal pressure-mediated transfection method (renal pressure method) as a kidney-specific in vivo gene delivery system. However, additional information on selecting other injection routes and applicable animals remains unclear. In this study, we selected renal arterial and ureteral injections as local administration routes and evaluated the characteristics of gene delivery such as efficacy, safety, and distribution in pressured kidney of rat. Immediately after the naked pDNA injection, via renal artery or ureter, the left kidney of the rat was pressured using a pressure controlling device. Transfection efficiency of the pressured kidney was about 100-fold higher than that of the injection only group in both administration routes. The optimal pressure intensity in the rat kidney was 1.2 N/cm^2^ for renal arterial injection and 0.9 N/cm^2^ for ureteral injection. We found that transgene expression site differs according to administration route: cortical fibroblasts and renal tubule in renal arterial injection and cortical and medullary tubule and medullary collecting duct in ureteral injection. This is the first report to demonstrate that the renal pressure method can also be effective, after renal arterial and ureteral injections, in rat kidney.

## 1. Introduction

Gene therapy is a potential approach for various incurable diseases. Naked plasmid DNA (pDNA) transfer is simple and less immunogenic, compared with other viral or non-viral vectors; but intravenous injection of naked pDNA shows low transfection efficiency [1]. Especially, gene delivery to the kidney is generally difficult due to the glomerular filtration barrier for size and charge. Several groups have reported that adding physical forces such as hydrodynamic [2,3] and electric [4,5] forces to the kidney enhances transgene expression.

Focused on pressure stimuli, we previously developed a renal pressure-mediated transfection (renal pressure method) [6,7] and suction-mediated transfection (renal suction method) methods [8,9] in mice. In these methods, direct pressure or suction after intravenous naked pDNA injection can achieve kidney-specific gene transfer without causing marked renal dysfunction in mice [6,7,8,9]. Although the direction of pressure stimulation is different between the renal pressure and suction methods, both methods induce tissue deformation [9,10].

More recently, we have optimized a multicolor deep imaging system, by a tissue clearing method and confocal microscopy, in various organs [10,11,12,13]. Using this imaging system, we have revealed that pDNA can be delivered to peritubular fibroblast of renal cortex in suctioned kidney, after systemic injection in mice [10]. However, gene delivery to cortical glomeruli, tubules, and medulla has not been achieved with intravenous routes because of the glomerular filtration barrier or basement membrane.

In kidney-targeting gene delivery, selection of administration route is important for determining the therapeutic target and in gene functional analysis. Additionally, local administration is desirable in order to minimize gene and nucleic acid distribution in systemic blood flow in future clinical settings [14,15]. So far, approaches from the renal artery [1,4,5,16] and vein [2,3] were mainly studied as local transfection to the kidney. As another approach, several groups have examined retrograde injection from the ureter or renal pelvis [1,5,15,17]. In view of the complicated structure of the nephron, we considered that the approach of retrograde injection from the ureter with pressure can theoretically achieve gene delivery into renal tubules by avoiding the glomerular filtration barrier. Since the glomeruli and tubules constituting the nephron are important for life support, accomplishment of gene delivery to these cells has a significant meaning. However, few studies have focused on combining these local administrations and pressure stimuli.

In this study, we selected two types of local administration routes: renal arterial and retrograde injections from ureter and attempted to elucidate the characteristics of gene delivery by the renal pressure method in rats. We firstly examined the efficacy and safety of this transfection method in the rat kidney. Secondly, we evaluated spatial distribution of the transgene expression using a multicolor deep imaging system, and determined transfected cells in the renal pressure method, with renal arterial and retrograde ureteral injections.

## 2. Materials and Methods

### 2.1. pDNA

pCMV-Luc, which contains a cytomegalovirus promoter and expresses firefly luciferase, was constructed as previously reported [18]. pCpG-free-LacZ was purchased from Invivogen (San Diego, CA, USA) and pZsGreen1-N1 was purchased from Clontech (Takara Bio Inc., Shiga, Japan). ^TM^Rhodamine-labeled pCMV-Luc was prepared using the Label IT-^TM^-Rhodamine Labeling Kit (Mirus Corp., Madison, WI, USA), according to the manufacturer’s instructions.

### 2.2. Animals

Seven-week-old male Sprague–Dawley (SD) rats were purchased from the Japan SLC Inc. (Shizuoka, Japan). All animal experiments were carried out in accordance with the Principles of Laboratory Animal Care, as adopted and promulgated by the US National Institutes of Health, and the guideline for animal experiments of Kyoto University or Nagasaki University. Project identification code: 2010-47 (2010) was approved by the Animal Research Committee, Kyoto University, and project identification code: 1812251497-2 (2019) was approved by the Institutional Animal Care and Use Committee of Nagasaki University.

### 2.3. pDNA Transfection

Rats were anesthetized with intraperitoneal injection of pentobarbital (50 mg/kg) or three types of mixed anesthetic agents as previously described [19], with slight modification (0.75 mg/kg of medetomidine, 4.0 mg/kg of midazolam, and 5.0 mg/kg of butorphanol). pDNA was administered to rats via two different routes, renal arterial and retrograde injections, from the ureter.

For renal arterial injection, the left kidney and renal artery were surgically exposed. After clamping of the left renal artery and vein with disposable vessel clips (TKS-1; Bear Medic, Chiba, Japan), 100 µg of pDNA in 200 µL of saline was injected into the left kidney, via the renal artery for 10 sec, using BD Ultra-Fine Insulin Syringes with a 30-gauge needle (Becton, Dickinson and Company, BD Franklin Lakes, NJ, USA). Immediately after injection, the left kidney was pressured using the thumb and index finger or a pressure-controlling device, meant for rats, for a period of 1 s. The duration of ischemia during renal arterial injection was shorter than 5 min. After transfection, the puncture was fixed with Aronalfa (Toagosei Co. Ltd., Tokyo, Japan), and the clamp released.

For retrograde injection from ureter, the left kidney and ureter were surgically exposed. After clamping the left renal vein with disposable vessel clips (TKS-1), 100 µg of pDNA in 100 µL of saline was injected into the left kidney via the ureter, using BD Ultra-Fine Insulin Syringes with a 30-gauge needle (Becton, Dickinson), for 5 sec. Immediately after injection, the left ureter was clamped and the left kidney was pressured in the same way. After renal pressure transfection, the puncture was fixed with Aronalfa, and the clamp released.

In the case of electroporation as a control experiment, electric pulses to the left kidney were delivered with a pair of 1 cm^2^ forceps-type electrodes immediately after intravenous injection of 100 µg of pDNA in 200 µL of saline. The electric pulse parameters were 100 ms pulse length, 6 pulses, 900 ms pulse intervals, and 75 V (CUY-21; Nepagene, Chiba, Japan) [20].

### 2.4. Controlling and Quantifying the Pressure Intensity

A previously reported pressure controlling device [7] was used with slight modification. As shown in Figure S1, the tip of the syringe fitting for mouse (Surface area: 1 cm^2^) was replaced with a larger one for rat kidney (Surface area: 2 cm^2^).

### 2.5. Luciferase Assay

Luciferase assay was performed as previously described [18]. Luciferase activities in the transfected kidney were normalized for protein concentration and measured using commercially available assay kit (Dojindo Molecular Technologies, Inc., Rockville, MD, USA).

### 2.6. Spatial Distribution of the Transgene Expression by Tissue Clearing Reagent

Twenty-four hours post pZsGreen1-N1 transfection by the renal pressure method, via left renal artery or left ureter, rats were perfused, via renal artery, with 50 mL of phosphate buffered saline (PBS) and 10 mL of 6.4 mM DiI (1,1′-dioctadecyl-3,3,3′,3′-tetramethylindocarbocyanine perchlorate) (42364; Sigma Aldrich, Inc., Saint Louis, MO, USA) solution in 5% glucose for nephron staining. The resected left kidneys were fixed with 4% paraformaldehyde and immersed in Sca*l*e SQ [21] reagent for 48 h. Three-dimensional images were acquired by confocal laser scanning microscopy (LSM710; Carl Zeiss Microimaging GmbH, Jena, Germany).

### 2.7. 5-Bromo-4-Chloro-3-Indolyl-β-D-Galactoside (X-Gal) Staining of Tissue Sections

Twenty-four hours post pCpG free-LacZ transfection via left renal artery or left ureter with/without renal pressure, rats were anesthetized and perfused via renal artery, with 50 mL of PBS and 50 mL of 0.5% glutaraldehyde in PBS. The left kidneys were resected and fixed with 0.5% glutaraldehyde in PBS, overnight at 4 °C. Then, the kidneys were embedded in optimal cutting temperature (OCT.) compound and 7 µm-thick cryostat sections stained with X-gal staining solution, for 4 h at 37 °C. The X-gal staining solution consisted of 1 mg/mL X-Gal (AG-CC1-0003; Adipogen Life Sciences, Inc., San Diego, CA, USA), 6 mM potassium ferricyanide, 6 mM potassium ferrocyanide, 2 mM MgCl_2_, 0.02% of IGEPAL CA-630, and 0.01% of deoxycholate, in PBS. The stained sections were observed under bright field, using a standard inverted microscope, AxioVert.A1 (Carl Zeiss, Oberkochen, Germany).

### 2.8. Observation of Fluorescent Conjugated pDNA of Tissue Sections

Ten micrograms of ^TM^Rhodamine-labeled pCMV-Luc solution in 200 µL (renal arterial injection) or 100 µL (ureteral injection) was injected, via the left renal artery or ureter, by the renal pressure method. Ten min after transfection, the left kidneys were perfused with 50 mL of PBS via the renal artery and resected. The collected kidneys were immediately embedded in OCT compound, and 7 µm-thick cryostat sections fixed with 4% paraformaldehyde (PFA) for 10 min. After incubation with 1% bovine serum albumin (BSA)-PBS, for 30 min at room temperature, the sections were stained with Alexa Fluor 488-phalloidin (A12379; Invitrogen, Carlsbad, CA, USA), for 20 min at room temperature. The stained sections were observed under a fluorescence microscope, BZ-X700 (Keyence Corp., Osaka, Japan)

### 2.9. Measurement of Serum Creatinine (Cre) and Blood Urea Nitrogen (BUN) Levels

Rats were transfected with pCMV-Luc via renal arterial or ureteral route, by the renal pressure method. To eliminate influence of the compensatory capacity of untreated kidneys on renal function, both kidneys were pressured after the left renal arterial or retrograde injections, from both sides of the ureter. For positive control, rats received a single administration of cisplatin (50 mg/kg) via their tail vein [22]. Three-hundred microliters of blood was collected from the tail vein on days 1, 2, 4, and 7 post transfection. The blood sample was coagulated for 1 h at room temperature and overnight at 4 °C, and serum was collected as the supernatant, following centrifugation (1000 *g*, 10 min, 4 °C) [6]. The serum creatinine and BUN levels were evaluated using commercially available assay kits (Wako Pure Chemicals Industries, Ltd., Osaka, Japan).

### 2.10. Measurement of N-Acetyl-β-D-Glucosaminidase (NAG) Levels

Rats were transfected with pCMV-Luc via renal artery or both sides of the ureter, and both kidneys pressured. As positive control, rats were intraperitoneally administered with Gentamicin (100 mg/kg/day) for 5 days [23,24]. Then, rats were housed in metabolic cages with food and water for 24 h. Twenty-four hour urine samples were collected on days 0, 1, 3, and 6 post administration. Collected samples were centrifuged for 5 min at 1000 *g* and the clear supernatants stored at 4 °C. *N*-acetyl-β-d-glucosaminidase (NAG) activity was measured using NAG test Shionogi (Shionogi & Co., Ltd., Osaka, Japan). Urine creatinine concentration was also measured, and NAG index was calculated from urine NAG activity and creatinine concentration.

### 2.11. Hematoxylin and Eosin (HE) Staining of Tissue Sections

Rats were transfected with pCMV-Luc via renal artery or both sides of the ureter, and both kidneys were pressured. For positive control, rats received intraperitoneal administration of Gentamicin (100 mg/kg/day). Twenty-four hours after transfection, the left kidneys were perfused with PBS and 4% PFA via the renal artery, and resected. The collected kidneys were paraffin-embedded. Paraffin sections of 5 µm thickness were stained with hematoxylin (Wako Pure Chemicals Industries, Ltd., Osaka, Japan) and eosin (Wako Pure Chemicals Industries, Ltd., Osaka, Japan). The stained sections were observed under bright field, using a fluorescence microscope, BZ-X700 (Keyence Corp., Osaka, Japan).

### 2.12. Statistical Analyses

Statistical significance was assessed by unpaired *t*-test for two groups. Multiple comparisons were performed, using Tukey’s or Dunnett’s tests with analysis of variance. *p* < 0.05 was considered statistically significant.

## 3. Results

### 3.1. Efficient Transgene Expression by the Renal Pressure Method via Local Administration from Renal Artery and Ureter in Rats

#### 3.1.1. Effect of Renal Pressure on Rat Kidney after Local Administration via Renal Artery or Ureter

**Figure 1:**
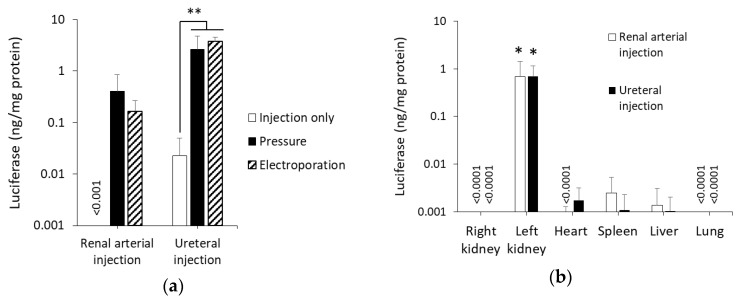
Effect of renal pressure on rat kidney after local administration via renal artery or ureter. Rats were injected with 100 µg of pCMV-Luc via the left renal artery or ureter, and immediately followed by pressing the left kidney for 1 s, with the thumb and index finger. Luciferase levels were determined 1 d post transfection. (**a**) Luciferase expression levels, with the renal pressure method (black column), were compared with electroporation (hatched column) and control groups (white column). Data are represented as mean + SD (*n* = 4–5). **p < 0.01 (Tukey’s test); (**b**) Luciferase expression in various organs by the renal pressure method via renal arterial route (white column) or ureteral route (black column). Data are represented as mean + SD (*n* = 4). *p < 0.05 (Tukey’s test).

To confirm whether the renal pressure method can be effective after renal arterial injection or ureteral injection, luciferase levels in the left kidney 1 d post administration were measured. In both local administration routes, luciferase levels of the pressured kidney increased by about 100-fold, compared with the injection only group (Figure 1a). Moreover, luciferase levels in the renal pressure group were as high as those in the electroporation method. Next, we evaluated transgene expression of various organs in the renal pressure method, via the renal arterial route or the ureteral route. Luciferase levels of the pressured kidney were significantly higher than those of other organs, including non-pressured kidney (Figure 1b).

#### 3.1.2. Effect of Pressure Intensity on Transgene Expression Levels

**Figure 2:**
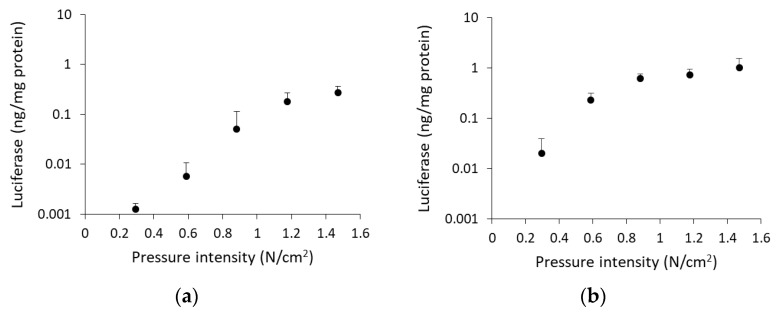
Effect of pressure intensity on transgene expression levels. Rats were injected with 100 µg of pCMV-Luc via (**a**) left renal artery or (**b**) left ureter, and immediately followed by pressing the left kidney using the pressure controlling device. Luciferase levels were determined 1 d post transfection. Data are represented as mean + SD (*n* = 4).

To analyze the relation between pressure intensity and transgene expression levels, we applied the pressure controlling device to rat kidney instead of gripping by the thumb and index finger. Luciferase levels increased with pressure intensity until 1.2 N/cm^2^ (renal arterial injection) or 0.9 N/cm^2^ (ureteral injection), but there was no further significant increase in luciferase activity at higher pressure intensities (Figure 2a,b). The luciferase levels at these pressure intensities corresponded to that of the finger-pressured group (Figure 1a). Therefore, the subsequent experiments were performed under the optimal pressure condition, using the pressure controlling device: 1.2 N/cm^2^ (renal arterial injection) and 0.9 N/cm^2^ (ureteral injection).

#### 3.1.3. Optimization of Injection Volume and pDNA Amount

**Figure 3:**
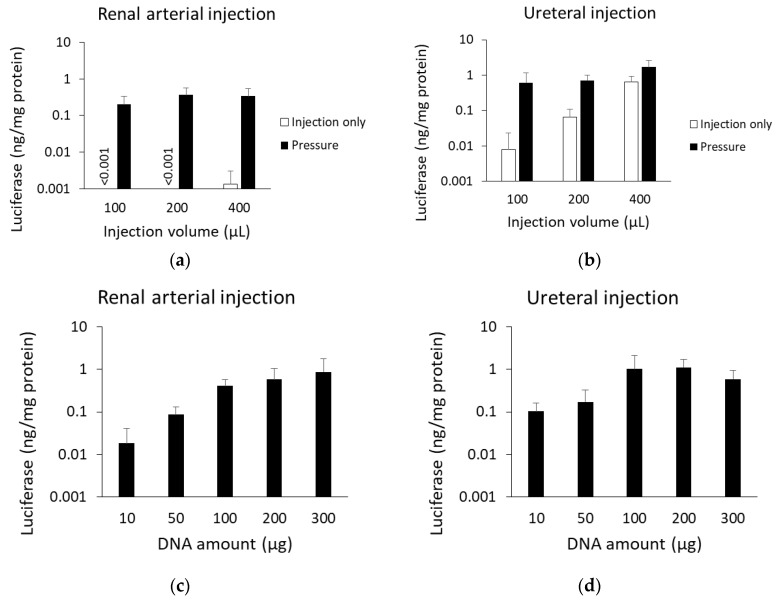
Optimization of injection volume and pDNA amount. (**a**,**b**) Rats were injected with 100 µg of pCMV-Luc in several injection volumes (100, 200, and 400 µL) via (**a**) left renal artery or (**b**) left ureter, and immediately followed by pressing the left kidney using the pressure controlling device. Effect of injection volume was determined in the renal pressure (black column) and injection only (white column) groups. (**c**,**d**) Rats were injected with several amounts of pCMV-Luc (10, 50, 100, 200, and 300 µg) in fixed volume via (**c**) left renal artery or (**d**) left ureter, and immediately followed by pressing the left kidney using the pressure controlling device. Effect of pDNA amount was evaluated with the standard injection volume (200 µL for renal arterial injection and 100 µL for ureteral injection). Luciferase levels were determined 1 d post transfection. Data are represented as mean + SD (*n* = 3–4).

To evaluate optimal conditions for gene transfer by the renal pressure method in rat, we performed comparative injection volume and pDNA amount analyses, using luciferase assay. At first, we assessed optimal volume between several injection volumes (100, 200, and 400 µL), with a fixed amount of pDNA (100 µg). In renal arterial injection, luciferase levels of the renal pressure group increased slightly but not significantly at 200 µL (Figure 3a). In ureteral injection, luciferase levels of the injection only group increased with increased injection volume, whereas luciferase levels of the renal pressure group were high at any injection volume (Figure 3b). Although the highest value was at 400 µL, the difference from the injection only group was largest at 100 µL. Then, renal arterial injection (200 µL) and ureteral injection (100 µL) volumes were selected as standards (Figure 3a,b). Next, we examined optimal amount of pDNA (10, 50, 100, 200, and 300 µg) in rat with the standard injection volume (200 µL for renal arterial and 100 µL for ureteral injections). In both injection routes, transgene expression levels were higher with increasing pDNA amount up to 100 µg. pDNA more than 200 µg did not increase transfection efficiency (Figure 3c,d). Unless otherwise noted, subsequent experiments were done with 100 µg of pDNA.

### 3.2. Distribution of pDNA Delivered by the Renal Pressure Method via Local Administration from Renal Artery or Ureter

#### 3.2.1. Spatial Distribution of Transgene Expression by the Renal Pressure Method via Local Administration from Renal Artery or Ureter

**Figure 4:**
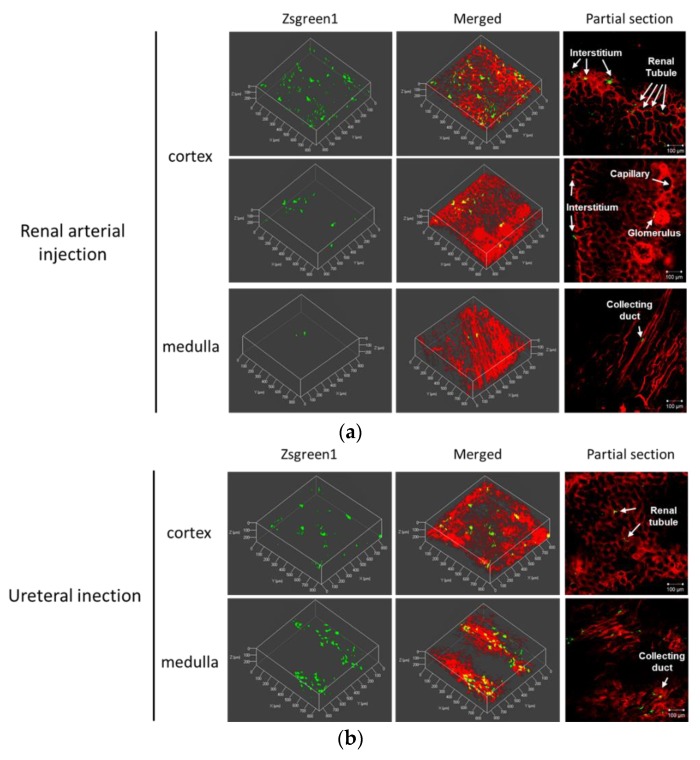
Spatial distribution of transgene expression by the renal pressure method via local administration from renal artery or ureter. Rats were injected with pZsGreen1-N1 via (**a**) left renal artery or (**b**) left ureter by the renal pressure method. Twenty-four hours post transfection, rats were perfused with DiI solution for nephron staining. The resected kidneys were immersed in Sca*l*e SQ reagent. The stained kidney was observed by confocal laser scanning microscopy. Objective lens: ×10 lens. Green: ZsGreen1 expression and Red: DiI-stained nephron. Scale bars represent 100 µm.

To clarify dispersibility of transgene expression in the kidney, the pressured kidney was prepared for a multicolor deep imaging system, by tissue clearing reagent Sca*l*e SQ. The fluorescent probe DiI perfused via renal artery stained blood vessels, renal tubules, and collecting ducts. Using the renal arterial injection route, ZsGreen1 expression was mainly observed in the cortex and located in interstitial region, renal tubule, and glomerulus (Figure 4a). The weighted colocalization coefficient of ZsGreen1 with DiI stained area was respectively 36.10 ± 15.84% in the cortex and 14.14 ± 13.28% in the medulla (Table S1a). The ratio of colocalized ZsGreen1 area /DiI stained area was respectively 0.91 ± 0.36% in the cortex and 0.80 ± 0.45% in the medulla (Table S1b). Using the ureteral injection route, ZsGreen1 expression was widely diffused from cortical renal tubule to medullary collecting duct (Figure 4b). The weighted colocalization coefficient of ZsGreen1 with DiI stained area was respectively 30.70 ± 19.33% in the cortex and 16.72 ± 6.64% in the medulla (Table S1a). The ratio of colocalized ZsGreen1 area /DiI stained area was respectively 0.49 ± 0.27% in the cortex and 0.86 ± 0.40% in the medulla (Table S1b).

#### 3.2.2. Evaluation of Transgene Expression Site after LacZ Transfection by the Renal Pressure Method via Local Administration from Renal Artery or Ureter.

**Figure 5:**
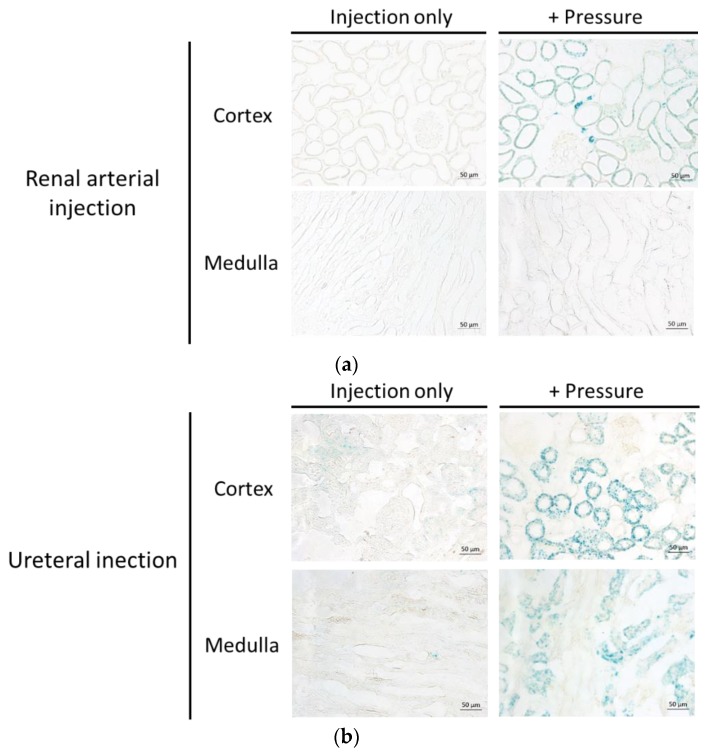
Evaluation of the transgene expression site after LacZ transfection by the renal pressure method via local administration from renal artery or ureter. Rats were injected with pCpG free-LacZ via (**a**) left renal artery or (**b**) left ureter, using the renal pressure method. Twenty-four hours post transfection, the kidney was collected and 7 µm-thick cryostat sections were stained with X-gal staining solution. The sections were observed under bright field. The renal pressure group (right) was compared with injection only group (left). Scale bars represent 50 µm.

To specify the transgene expression site, X-gal staining of the frozen section was performed after LacZ transfection by the renal pressure method. In the renal arterial injection group, β-galactosidase expression was localized in the cortex. The transgene expression was exhibited markedly in peritubular fibroblast, and partly in renal tubules and Bowman’s capsule (Figure 5a). In the ureteral injection group, β-galactosidase was observed in both the cortex and medulla. The transgene expression was distributed in proximal tubular and collecting duct cells (Figure 5b).

#### 3.2.3. Distribution of Fluorescent-Labeled pDNA by the Renal Pressure Method via Local Administration from Renal Artery or Ureter

**Figure 6:**
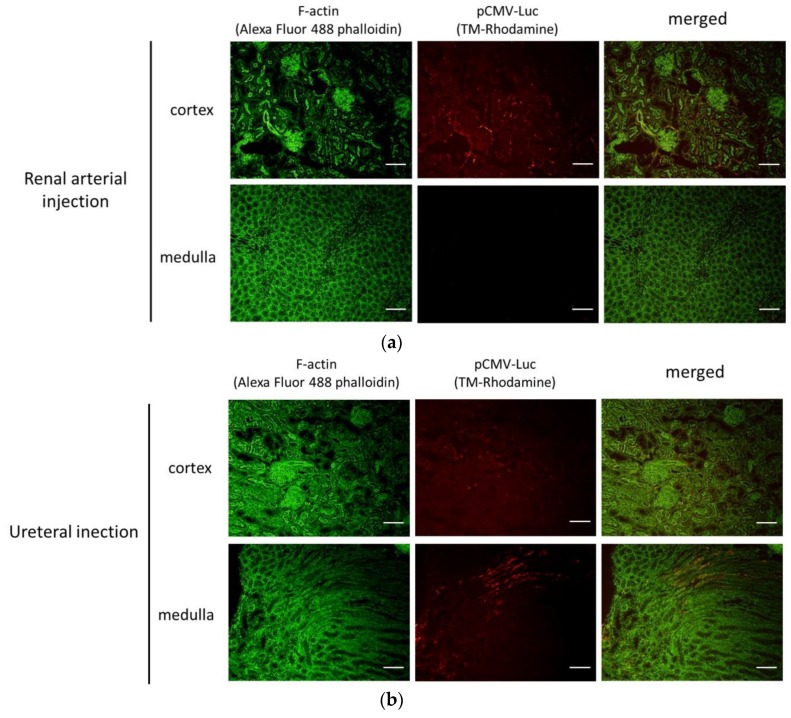
Distribution of fluorescent-labeled pDNA by the renal pressure method via local administration from renal artery or ureter. Rats were injected with ^TM^Rhodamine-labeled pCMV-Luc via (**a**) left renal artery or (**b**) left ureter by the renal pressure method. Ten min post transfection, the kidney was collected, and 7 µm-thick cryostat sections stained, using Alexa Fluor 488 phalloidin. The stained sections were observed using a fluorescent microscope. Objective lens: ×10 lens. Green: F-actin stained by Alexa Fluor 488 phalloidin and Red: ^TM^Rhodamine-labeled pCMV-Luc. Scale bars represent 100 µm.

pDNA distribution was evaluated 10 min post renal pressure transfection via the renal artery or ureter. In the renal arterial injection group, ^TM^Rhodamine-labeled pCMV-Luc was observed in the cortex, but not in the medulla. In the cortex, ^TM^Rhodamine-labeled pDNA was distributed in the interstitial area, renal tubule, and Bowman’s capsule (Figure 6a). On the other hand, ^TM^Rhodamine-labeled pCMV-Luc was observed in both the cortex and the medulla. Fluorescence was observed in the interstitial area, proximal and distal tubules, and collecting duct (Figure 6b).

### 3.3. Assessment of Renal Function after Renal Pressure Transfection

#### 3.3.1. Glomerular Filtration Rate

**Figure 7:**
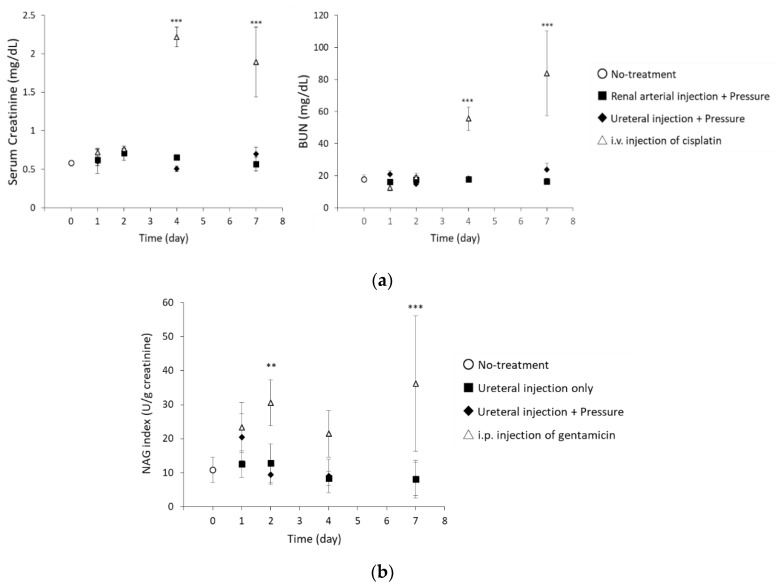
Evaluation of biomarkers for renal damage. (**a**) Serum creatinine and blood urea nitrogen (BUN) concentrations on days 1, 2, 4, and 7 post transfection by the renal pressure method. Both kidneys in the rat were pressured after pCMV-Luc injection via left renal artery (■) or both ureters (♦). As negative control, no-treatment group (○) was measured. Single intravenous injection of cisplatin (△) was selected as positive control. Data are represented as mean + SD (*n* = 3–5). ***p < 0.001 vs. no-treatment (Dunnett’s test). (**b**) Urine NAG assessment on days 1, 2, 4, and 7 post ureteral pDNA injection. Rats were administered with pCMV-Luc via both ureters (■), immediately followed by pressing both kidneys (♦). As negative control, no-treatment group (○) was assessed. As positive control, rats were intraperitoneally injected with gentamicin (100 mg/kg/day) for 5 days (△). Twenty-four hour urine samples were collected on days 0, 1, 3, and 6 post administration, and urine NAG activity and creatinine concentrations measured. NAG index was calculated as NAG/Creatinine (U/g creatinine). Data are represented as mean ± SD (*n* = 4–5). **p < 0.01, ***p < 0.001 vs. no-treatment (Dunnett’s test).

To determine whether the renal pressure method induced possible renal dysfunction, glomerular filtration rate was assessed. In both administration routes, serum creatinine and BUN levels were not significantly different from those of the no-treatment group at any time points (Figure 7a). Thus, renal pressure did not affect glomerular function.

#### 3.3.2. Assessment of Tubular Damage

Since retrograde ureteral injection could cause tubular damage, urine NAG activity was assessed in ureteral injection route with/without pressing both kidneys. The NAG level in the renal pressure group was higher than that in the no-treatment group, 1 day post treatment, while NAG levels in the injection only group was not different from the no-treatment group (Figure 7b). Two days post transfection, by the renal pressure method, NAG level returned to the normal range (Figure 7b).

#### 3.3.3. Histological Assessment of Renal Damage

**Figure 8:**
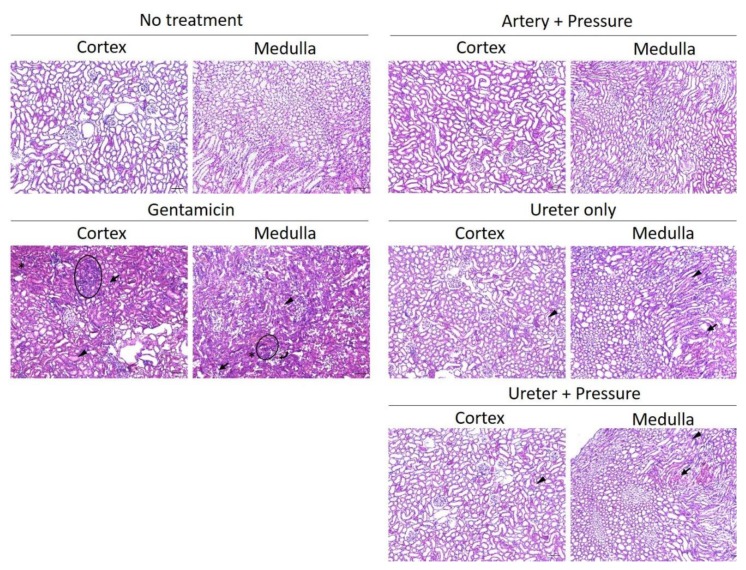
Histological assessment of renal damage. Rats were injected with pCMV-Luc via left renal artery or both ureters, using the renal pressure method. As negative control, the no-treatment group was assessed. As positive control, rats were intraperitoneally injected with gentamicin (100 mg/kg/day). The kidney was resected 24 h post transfection, and 7 µm-thick paraffin sections prepared for HE staining. Arrows: hemorrhages. Arrowhead: tubular dilatation. Curved arrow: hyaline casts. Circle area: leucocytes infiltration. Asterisk: Necrotic tubular epithelial cells. Objective lens: ×10 lens. Scale bars represent 100 µm.

Histological change was assessed by HE staining of paraffin sections 24 h post transfection. In renal arterial injection, renal pressure did not induce morphological changes in both the cortex and medulla. In ureteral injection, hemorrhages were mainly observed in the medulla, regardless of, presence or absence renal pressure. Although tubular dilatation was partly observed in the ureteral injection group, there were less signs of hyaline casts, necrosis, and mononuclear infiltration, compared with the gentamicin group (Figure 8).

## 4. Discussion

Kidney-targeted naked gene transfection, using physical pressure, has an advantage from the standpoint of simple component over other viral vectors and synthetic carriers [6,8,10]. So far, we developed the renal pressure [6,7] and suction [8,9] methods, using direct pressure to the kidney. However, the application was limited only to intravenous injection route, and additional information on selecting other injection routes and applicable animals remain unclear. Therefore, we attempted to develop the renal pressure method for rats using local administration from renal artery and ureter. As shown in Figure 1a,b, transgene expression in the pressured kidney showed tissue specificity and high efficiency as the electroporation method, which is reported to achieve efficient transgene transfection in rat kidney [4,5,25]. This is the first report to apply the renal pressure method in rats and shows that the renal pressure method can also be effective after renal arterial and ureteral injections.

We have reported that the key factor for efficient gene transfer, using pressure and suction, is inducing tissue deformation [9,10,26,27], and the minimal intensity of pressure or suction was often different in each organ [7,9,26]. Because of the differences in tissue hardness and volume capacity, pressure intensity needs to be optimized for rat kidney. The pressure intensity measured by the pressure controlling device for rats plateaued at 1.2 N/cm^2^ for renal arterial injection and 0.9 N/cm^2^ for ureteral injection (Figure 2a,b). Compared with the optimal pressure intensity for mouse kidney (0.59 N/cm^2^) via vascular system [7], that for rat kidney was twice as much. Additionally, the optimal injection volume was evaluated. In renal arterial injection, injection volume did not significantly affect transfection efficiency, by the renal pressure method (Figure 3a). On the other hand, in ureteral injection, without pressing kidney, transgene expression increased in proportion to injection volume (Figure 3b). Several groups have reported that retrograde injection from ureter or renal pelvis, without physical stimuli, can achieve efficient gene transfer to the kidney [1,7,28,29]. Moreover, Woodard et al. have reported retrograde injection via renal pelvis, using hydrodynamic principle of fluid pressure and speed [17,30]. Therefore, the controlling factor of the renal pressure method via ureteral injection is suggested to be hydrodynamic force by injection speed and mechanical stress by subsequent tissue deformation. This might account for why ureteral injection required lower pressure intensity than renal arterial injection. These data suggest that optimizing the pressure intensity according to animal species, tissue, and injection route is important to expand the application of the tissue pressure method.

For effective gene therapy, it is important to transfer therapeutic gene into target site. In kidney-targeted gene delivery, the gene is accessible by different routes, including renal artery, parenchyma, ureter, or renal pelvis; selecting administration routes can determine the transfected sites [1,29,31]. Firstly, we assessed the distribution of transgene expression and fluorescent-labeled pDNA in the renal pressure method, after renal arterial injection. As shown in Figure 5a and Figure 6a, using the renal arterial injection route, transgene expression and fluorescent-labeled pDNA were observed mainly in interstitial fibroblasts and partly in renal tubules and Bowman’s capsule. Moreover, spatial distribution study revealed that transgene expression was abundant in renal cortex (Figure 4a). In terms of transgene expression in interstitial fibroblasts, this result is in accord with our previous report of the renal suction method after intravenous injection [10]. In contrast, this is incongruent with the result that the electroporation method, after renal arterial injection, transfects genes and nucleic acids to glomeruli [4,5,16]. The underlying mechanism of the renal pressure method was not well investigated in this study. However, the different results between renal pressure and electroporation methods raise the possibility that renal pressure cannot enter endothelial cells but may increase the permeability of basement membranes and allow pDNA entry into interstitial fibroblasts and epithelial cells of the tubules and Bowman’s capsule. Taken together, renal pressure after renal arterial injection can efficiently transfect gene into interstitial fibroblasts and epithelial cells in the cortex.

As the other injection route, several groups have reported that retrograde injection from ureter or renal pelvis is a promising route to target cortical and medullary renal tubules, using viral and non-viral vectors [1,15,17,29]. To confirm whether it is possible in combination with renal pressure, we evaluated distribution of transgene expression and fluorescent-labeled pDNA, using the renal pressure method, after ureteral injection. As shown in Figure 5b, even at low injection volume, slight transgene expression was observed in the ureteral injection only group; transgene expression was distributed in proximal tubular and collecting duct cells after renal pressure transfection. Additionally, results of pDNA distribution supported the result of transgene expression site (Figure 6b). The appearance of transgene expression is similar to those reported by other groups [1,15,17]. Moreover, multicolor deep imaging showed transgene expression widely diffused in cortical and medullary tubules and medullary collecting ducts by the renal pressure method (Figure 4b). These results support the view that the renal pressure method via ureteral injection is an effective and promising approach to gene delivery to renal tubules and collecting duct.

Although our previous results have shown that renal pressure or suction induced little renal toxicity [6,9], we considered the influence of temporary ischemia during renal arterial injection or hydrodynamic force during retrograde ureteral injection. Here, we evaluated the renal pressure method-induced renal damage, after local administration, by measuring renal injury markers and histological assessment. As shown in Figure 7a and Figure 8, renal functional analysis showed that renal pressure after renal arterial injection did not change glomerular filtration capacity and morphology. In spite of many reports about transfection efficiency or transgene distribution in retrograde ureteral injection, few reports are available on its renal damage [1,5,15]. In ureteral injection route, urine NAG activity transiently increased 1 day post renal pressure transfection, but it returned to normal levels within 2 days (Figure 7b). Histological assessment indicated that ureteral injection, regardless of the presence of absence renal pressure, induced hemorrhages and slight tubular dysfunction in the medulla (Figure 8). This histological change was similar to those reported by Woodard et al. [17]. These results suggest that renal pressure induces little nephrotoxicity, after renal arterial injection and causes transient tubular injury within 2 days after ureteral injection.

Administration of pDNA is considered safe, especially when employed at a low dose [32]. Clinical study of pDNA has been conducted at 4–16 mg by intra-muscular injection [33,34]. In our previous study in mice, we used the optimal pDNA dose 100 µg per mouse, by the renal pressure [6,7] and suction [9,10] methods, via systemic administration. Although it depends on body weight, the pDNA dose in mice corresponds to 4 mg/kg weight, which is an excessive dose. To achieve gene transfection safely and at lower cost, the pDNA dose needs to be reduced. As shown in Figure 3c,d, a pDNA dose of 100 µg per rat was enough for efficient gene transfection by the renal pressure method in both local injection routes, and it was almost equal to 0.4 mg/kg weight. Therefore, selecting local administration route from renal artery and ureter can reduce the pDNA dose, by about one-tenth in the renal pressure method, by minimizing systemic diffusion.

Local fibroblast expansion and epithelial-to-mesenchymal transition has been reported to play an important role in renal fibrosis progression [35,36]. As shown in Figure 4 and Figure 5, this study showed that the renal pressure method can effectively deliver gene to tubular epithelial cells (renal arterial and ureteral injections) and interstitial fibroblasts (renal arterial injection). Although the duration of transgene expression needs to be further improved by selecting the various vectors such as CpG free plasmid [10], φC31 integrase expression plasmid [37], and *piggyBac* transposon vector [17], the renal pressure method via local administration routes might be applied for renal fibrosis treatment using transient expression of growth factors. So far, therapeutic effect against renal fibrosis has been studied by gene or microRNA replacement therapy [38,39,40]. Recently, messenger RNA has attracted attention as a novel drug in terms of efficiency and quickness of protein expression and safety [41,42,43]. To achieve stable mRNA delivery, the renal pressure method via local administration route might be one of the good applications in future study.

## 5. Conclusions

Firstly, we demonstrated that the renal pressure method can be applied to rat kidney via local administration from renal artery and ureter. The optimal pressure intensity was 1.2 N/cm^2^ for renal arterial injection and 0.9 N/cm^2^ for ureteral injection in rat kidney, suggesting that a systematic application of the renal pressure method is important for clinical study to optimize pressure intensity, according to animal species, tissue, and injection route. Spatial distribution study revealed that transgene expression widely diffused in renal cortex for renal arterial injection and in renal cortex and medulla for ureteral injection. Interestingly, we found that transgene expression site differs according to administration route: cortical fibroblasts and renal tubule in renal arterial injection and cortical and medullary tubule and medullary collecting duct in ureteral injection. We believe that the renal pressure method via local administration can be a promising approach to develop novel gene therapy and in functional analysis using transient gene expression.

## Supplementary Materials

The following are available online at https://www.mdpi.com/1999-4923/12/2/114/s1, Figure S1: Pressure controlling device for rats. Table S1: Quantitative colocalization analysis of ZsGreen1 with DiI stained area. (a) The mean weighted colocalization coefficients of ZsGreen1 of five z-stacks per group were analyzed using LSM710 software (Carl Zeiss Microimaging GmbH, Jena, Germany). (b) The ratio of ZsGreen1 area relative to DiI stained area was calculated as ZsGreen1 area (μm × μm) /DiI stained area (μm × μm) (%) of ZsGreen1 and colocalized ZsGreen1.
