# Supplementary material for: Effects of Tissue Pressure on Transgene Expression Characteristics via Renal Local Administration Routes from Ureter or Renal Artery in the Rat Kidney
Source: Pharmaceutics. 2020 Feb 1;12(2):114. doi: 10.3390/pharmaceutics12020114 (PMC7076412; doi:10.3390/pharmaceutics12020114)
Supplement: Supplementary file 1 [file pharmaceutics-12-00114-s001.pdf]

pharmaceutics

Supplementary Materials: Effects of Tissue Pressure on Transgene Expression Characteristics via Renal Local Administration Routes from Ureter or Renal Artery in the Rat Kidney

Natsuko Oyama, Haruyuki Takahashi, Maho Kawaguchi, Hirotaka Miyamoto, Koyo Nishida, Masako Tsurumaru, Mikiro Nakashima, Fumiyoshi Yamashita, Mitsuru Hashida and Shigeru Kawakami

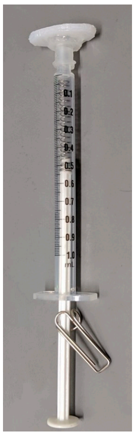

Figure S1. Pressure controlling device for rats.

Table S1. Quantitative colocalization analysis of ZsGreen1 with DiI stained area. (a) The mean weighted colocalization coefficients of ZsGreen1 of five z-stacks per group were analyzed using LSM710 software (Carl Zeiss Microimaging GmbH, Jena, Germany). (b) The ratio of ZsGreen1 area relative to DiI stained area was calculated as ZsGreen1 area (μm × μm) /DiI stained area (μm × μm) (%) of ZsGreen1 and colocalized ZsGreen1.

(a)

The mean weighted Colocalization Coefficients (%)

Renal arterial injection		Ureteral injection	
Cortex	Medulla	Cortex	Medulla
36.10 ± 15.84	14.14 ± 13.28	30.70 ± 19.33	16.72 ± 6.64

(b)

ZsGreen1 area /DiI stained area (%)

	Renal arterial injection		Ureteral injection	
	Cortex	Medulla	Cortex	Medulla
ZsGreen1	4.02 ± 2.43	0.27 ± 0.26	1.30 ± 0.53	4.35 ± 2.06
Colocalized ZsGreen1	0.91 ± 0.36	0.80 ± 0.45	0.49 ± 0.27	0.86 ± 0.40
